# CDK-Taverna: an open workflow environment for cheminformatics

**DOI:** 10.1186/1471-2105-11-159

**Published:** 2010-03-29

**Authors:** Thomas Kuhn, Egon L Willighagen, Achim Zielesny, Christoph Steinbeck

**Affiliations:** 1 Institute for Bioinformatics and Cheminformatics, University of Applied Sciences of Gelsenkirchen, Recklinghausen, Germany; 2 Department of Pharmaceutical Biosciences, Uppsala University, Uppsala, Sweden; 3 Chemoinformatics and Metabolism, European Bioinformatics Institute (EBI), Cambridge, UK

## Abstract

**Background:**

Small molecules are of increasing interest for bioinformatics in areas such as metabolomics and drug discovery. The recent release of large open access chemistry databases generates a demand for flexible tools to process them and discover new knowledge. To freely support open science based on these data resources, it is desirable for the processing tools to be open source and available for everyone.

**Results:**

Here we describe a novel combination of the workflow engine Taverna and the cheminformatics library Chemistry Development Kit (CDK) resulting in a open source workflow solution for cheminformatics. We have implemented more than 160 different workers to handle specific cheminformatics tasks. We describe the applications of CDK-Taverna in various usage scenarios.

**Conclusions:**

The combination of the workflow engine Taverna and the Chemistry Development Kit provides the first open source cheminformatics workflow solution for the biosciences. With the Taverna-community working towards a more powerful workflow engine and a more user-friendly user interface, CDK-Taverna has the potential to become a free alternative to existing proprietary workflow tools.

## Background

Small molecules are of increasing interest for bioinformatics in areas such as metabolomics and drug discovery. The recent release of large open chemistry databases into the public domain [1-4] calls for flexible, open toolkits to process them. These databases and tools will, for the first time, create opportunities for academia and third-world countries to perform state-of-the-art open drug discovery and translational research - endeavors so far a domain of the pharmaceutical industry. Commonly used in this context are workflow engines for cheminformatics, where numerous recurring tasks can be automated, including tasks for

• chemical data filtering, transformation, curation and migration workflows

• chemical documentation and information retrieval related workflows (structures, reactions, pharmacophores, object relational data etc.)

• data analysis workflows (statistics and clustering/machine learning for QSAR, diversity analysis etc.)

The workflow paradigm allows scientists to flexibly create generic workflows using different kinds of data sources, filters and algorithms, which can later be adapted to changing needs. In order to achieve this, library methods are encapsulated in Lego™-like building blocks which can be manipulated with a mouse or any pointing device in a graphical environment, relieving the scientist from the need to learn a programming language. Building blocks are connected by data pipelines to enable data flow between them, which is why *pipelining *is often used interchangeably for *workflow*. Workflows are increasingly used in cheminformatics research [5,6].

Existing proprietary or semi-proprietary implementations of the workflow or pipeline paradigm in molecular informatics include Pipeline Pilot [7] from SciTegic, a subsidiary of Accelrys or the InforSense platform from InforSense [8]. Both are commercially well established but closed source products with a large variety of different functionality. KNIME [9] is a modular data exploration platform which uses a dual licensing model with the Aladdin free public license. It is developed by the group of Michael Berthold at the University of Konstanz, Germany. KNIME is based on the open source Eclipse platform. An overview of workflow systems in life sciences was recently given by Tiwari *et al. *[10].

In 2005 we started to integrate our open source cheminformatics library, the Chemistry Development Kit (CDK) [11,12] with Taverna [13-15], a workflow environment with an extensible architecture, to produce CDK-Taverna, the first completely free [16] workflow solution for cheminformatics, which we present here.

It makes additional use of other open source components such as Bioclipse [17] for visualization of workflow results, and Pgchem::tigress [18] as an interface to the database back-end for storage of large data sets.

## Implementation

The here introduced CDK-Taverna plugin takes advantage of the plug-in detection manager of Taverna for its installation. This manager requires a plug-in description XML file containing a plug-in name, a version number, a target Taverna version number, a repository location and a Maven-like Java package description, all provided by the plug-in's installation website: http://www.cdk-taverna.de/plugin/.

After adding this URL, the manager presents all available plug-in versions graphically to the user. In order to install the CDK-Taverna plug-in the user selects the desired version after which all necessary Java libraries are installed on-the-fly from the given installation website.

The CDK-Taverna plug-in is written in Java is published under the GNU Lesser General Public License (LGPL). Version 0.5.1.1 uses CDK revision 12084. Like Taverna itself the CDK-Taverna plug-in uses Maven 2 [19] as a build system.

To integrate the CDK functionality, the plug-in makes use of the extension points provided by Taverna allowing dynamic discovery of the provided functionality. The following sections describe what extension points are used, and how molecular data is represented when flowing through the workflow.

### Taverna's extension points

Taverna allows the execution of workflows linking together heterogeneous open services, applications or databases (remote or local, private or public, third-party or home-grown) [20]. For the integration of these different resource types Taverna provides various interfaces and protocols for its extension. For example, it allows for easy access to webservices through WSDL [21] and SOAP [22].

The CDK-Taverna plug-in, on the other hand, uses Taverna's local extension mechanism. For local extensions, Taverna provides a list of different Service Provider Interfaces (SPI), as given in Table 1. CDK-Taverna implements several of these, integrating CDK functionality as so-called Local Workers which run on the same machine as the Taverna installation. Full JavaDoc documentation of the plug-in's source code is available at http://cdk.sourceforge.net/cdk-taverna/api/.

**Table 1:** List of available Service Provider Interfaces that can be used to create plug-ins for Taverna to provide additional functionality.

Interfaces
org.embl.ebi.escience.scuflworkers.java.LocalWorker

net.sf.taverna.perspectives.PerspectiveSPI

org.embl.ebi.escience.scuflui.spi.ProcessorActionSPI

org.embl.ebi.escience.scuflworkers.ProcessorInfoBean

org.embl.ebi.escience.scuflui.spi.RendererSPI

org.embl.ebi.escience.scuflui.spi.ResultMapSaveSPI

org.embl.ebi.escience.scuflui.workbench.scavenger.spi.ScavengerActionSPI

org.embl.ebi.escience.scuflworkers.ScavengerHelper

org.embl.ebi.escience.scuflui.workbench.Scavenger

org.embl.ebi.escience.scuflui.actions.ScuflModelActionSPI

org.embl.ebi.escience.scuflui.spi.UIComponentFactorySPI

All workers in CDK-Taverna implement the CDKLocalWorker interface. It is used for the detection of workers by the CDKScavenger class which itself implements the Taverna SPI org.embl.ebi.escience.scuflui.workbench.Scavenger interface. Adding user interfaces for some of the workers requires an extension of the AbstractCDKProcessorAction which again implements the Taverna SPI org.embl.ebi.escience.scuflui.spi.ProcessorActionSPI. The use of this SPI allows the addition of, for example, file chooser dialogs for workers like file reader or writer.

### The anatomy of a CDK-Taverna worker

To create a CDK-Taverna worker the Java class of this worker has to implement the CDKLocalWorker Interface. This interface defines that every worker has to define the following methods:

public Map <String, DataThing> execute(Map String, DataThing inputMap)

   throws TaskExecutionException;

public String[] inputNames();

public String[] inputTypes();

public String[] outputNames();

public String[] outputTypes();

The method inputNames and outputNames return the names of the ports of each worker whereas the inputTypes and outputTypes methods return the names for the Java object types with its package declaration e.g. java/java.util.List for a List. Within CDK-Taverna chemical structures are passed around using the Java object java/org.openscience.cdk.applications.taverna.CMLChemfile

## Results

The CDK-Taverna plug-in currently provides 164 different cheminformatics workers. The fields of application of these workers are described in Table 2. These include workers for input and output (I/O) of various chemical files and line notations formats, databases, and descriptors for atoms, bonds and molecules. The miscellaneous workers are e.g. a substructure filter, an aromaticity detector, an atom typer or a reaction enumerator. Some of the workers are outlined as part of example workflows described below (for a complete list see [23] or http://cdk.sourceforge.net/cdk-taverna/workers.html). In the following, we outline the application of CDK-Taverna with selected workflow scenarios. A larger list of workflows is available from MyExperiment.org [24].

**Table 2:** The worker allocation of CDK-Taverna by function.

Workers by function	Number of workers	Examples
File I/O	15	SDFParser, CML Reader & Writer

SMILES tools	2	SMILES Parser, SMILES Writer

InChI parser	2	InChI Parser, InChI Generator

Database I/O	7	Insert Molecules Into Database, Read Molecules From Database

Molecular descriptors	42	AtomCount & LargestChain

Atom descriptors	27	AtomHybridization & BondsToAtom

Bond descriptors	6	PartialPiCharge

Clustering	13	K-Means, ART 2-A Classification

Miscellaneous	50	Substructure Search, Reaction Enumeration

### Iteration over large data sets

Cheminformatics by definition deals with the discovery of chemical knowledge from large data collections. Because these data sources are usually too large to be loaded into memory as a whole, it is needed to loop over all data entries to process them one by one. Unfortunately, the architecture of Taverna 1.7 does not support such loops. CDK-Taverna, therefore, provides workers which act like FOR or WHILE loops, making use of Taverna's iteration-and-retry mechanism to allow workflows to process large data sets.

### Database I/O

For database support the CDK-Taverna project uses the PostgreSQL [25] relational database management system (RDBMS) with the open source Pgchem::tigress [18] extension. This combination allows storage and fast retrieval of up to a million molecules without running into memory limitations. The Pgchem::tigress extension uses an implementation of the Generalized Search Tree (GiST) [26] of the PostgreSQL database. CDK-Taverna can use a local installation of the PostgreSQL Database with the Pgchem::tigress extension or can connect to a remote instance.

#### Scenario 1: Substructure Search

**Figure 1:**
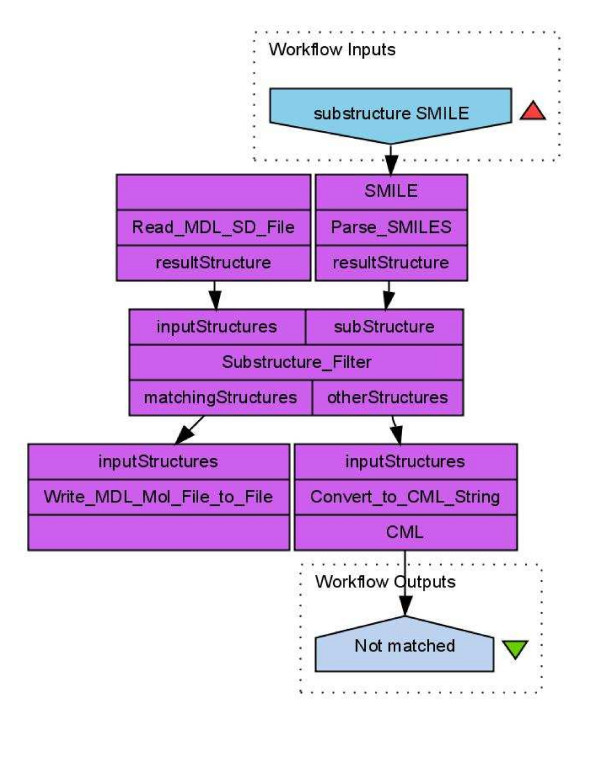
**Workflow performing a topological substructure search (Scenario 1) on molecules from a MDL SDfile **[35]. The input of this workflow is a SMILES string which represents the substructure.

We may want to design workflows for performing substructure searches in different ways depending on the type of input. In a first example the substructure workflow performs a topological substructure search on a list of given molecules and a given molecular substructure (see Figure 1). The workflow inputs are a molecular substructure represented in the SMILES line notation [27] and a list of structures stored in a MDL SDfile [28]. The structures which match the substructure are stored as MDL Molfiles [28]. The non-matching structures are converted into the Chemical Markup Language (CML) file format [29-31]. This small example workflow already combines four different molecular structure representations and the use of a topological substructure filter.

**Figure 2:**
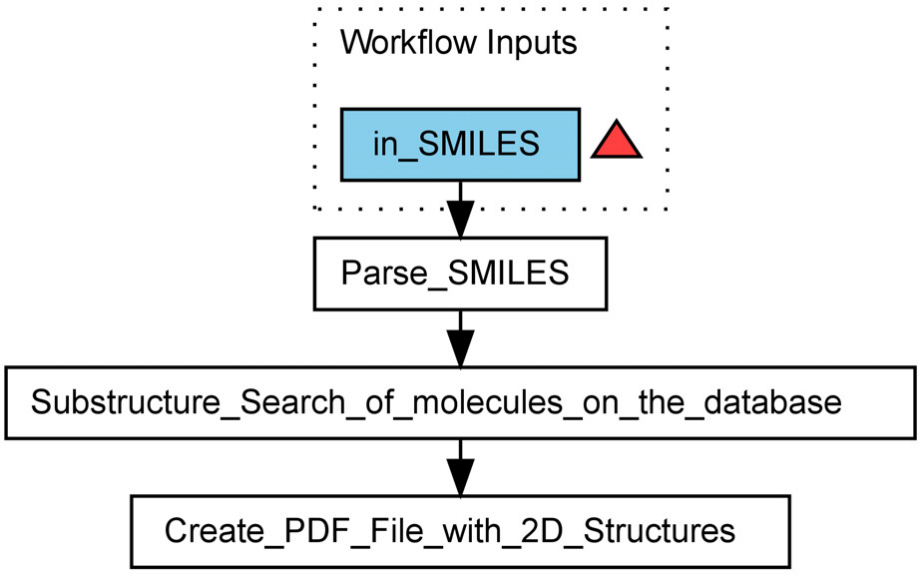
**Workflow performing a substructure search (Scenario 1) in a database **[36]. The substructure is defined with a SMILES string. The output is a PDF file with a tabular view of the molecules from the database containing the substructure.

A related workflow performs a substructure search directly on a database: It uses functionality provided by the Pgchem extension of the PostgreSQL database. This extension allows the use of SQL commands to perform a substructure search. A demonstration is given with the workflow in Figure 2. The molecules containing the substructure are reported in tabular form in a PDF file.

#### Scenario 2: Descriptor Calculation

**Figure 3:**
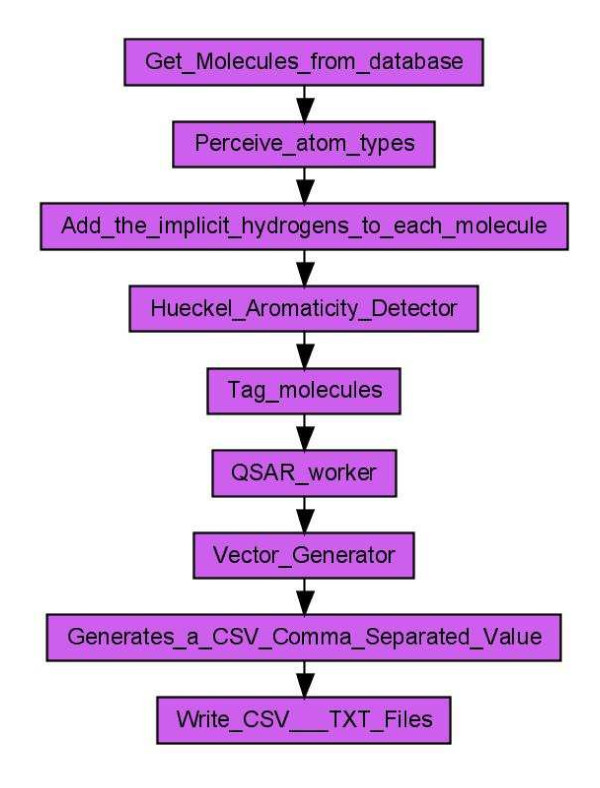
**Workflow calculating various QSAR descriptors (Scenario 2) for molecules from a PostgreSQL database**. The results of the calculation are stored in a CSV file.

**Figure 4:**
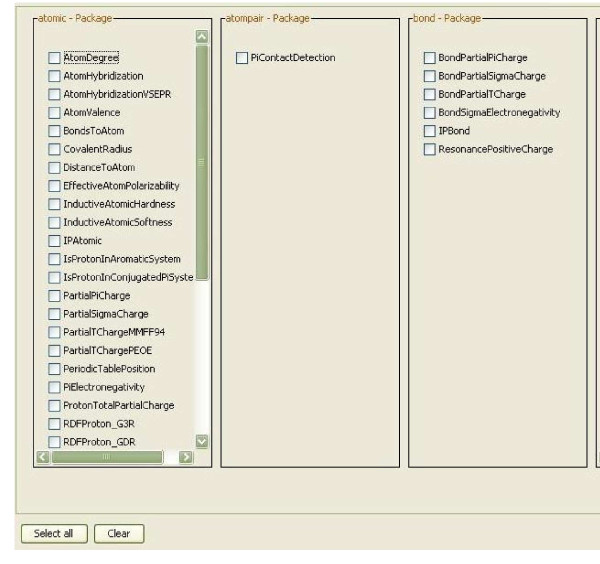
**User interface to select QSAR descriptors to be calculated for each molecule during the execution of the descriptor calculation workflow shown in Figure 3**.

The descriptor calculation workflow, depicted in Figure 3, starts with loading its molecules from a PostgreSQL database. The recognition of the atom types is the next step, followed by the addition of implicit hydrogens for each molecule as well as the detection of Hückel aromaticity. Each molecule is tagged for the descriptor calculation process. The tagging of the molecules is used to add a universal identifier to each molecule. This allows the identification of the corresponding Quantitative Structure-Activity Relationship (QSAR) descriptor values within the table of calculated descriptor values. The QSAR worker provides a user interface based selection of multiple QSAR descriptors (see Figure 4) for the calculation of a molecule's property vector based on the CDK.

**Figure 5:**
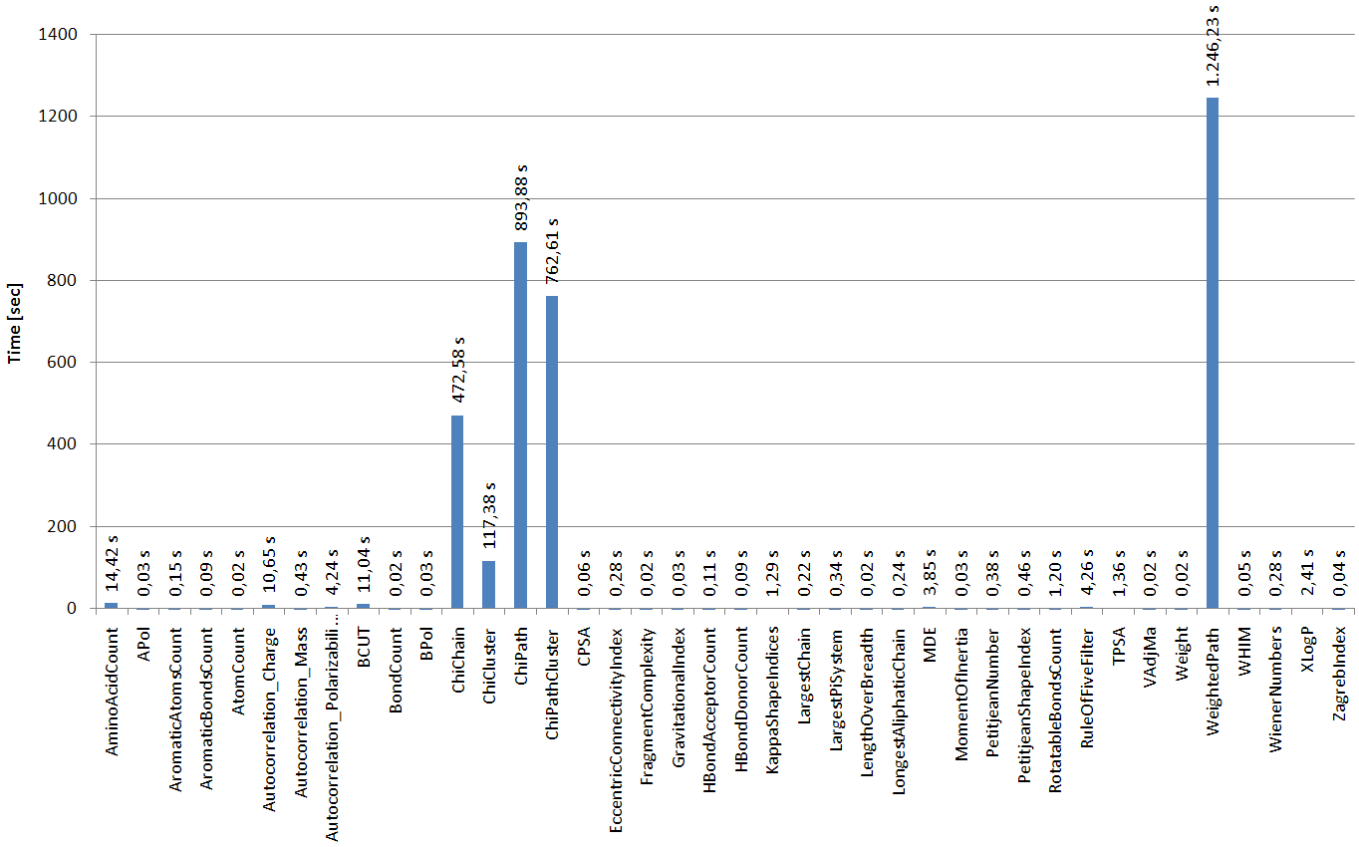
**Overview of the time needed to calculate different molecular descriptors for 1000 molecules **[37].

The result of the workflow is a comma separated value (CSV) text file which contains the ID of the molecule and the calculate property values. The property vectors may then be used for statistical analysis, clustering or machine learning purposes. With the PostgreSQL Database back-end CDK-Taverna is able to calculate a large number of descriptors for many thousand of molecules in a reasonable time (see Figure 5).

#### Scenario 3: Iterative Descriptor Calculation

**Figure 6:**
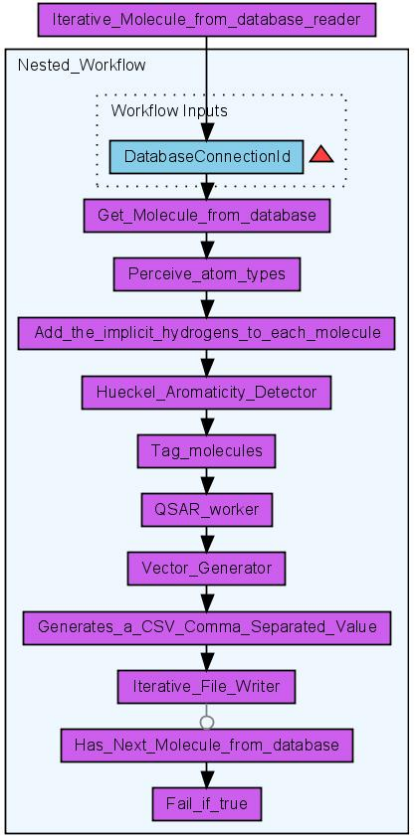
**Workflow iteratively calculating different QSAR descriptors (Scenario 3) for molecules loaded from a PostgreSQL database **[38]. The results are stored in a CSV file.

The iterative descriptor calculation workflow is a *work-around *which allows the treatment of hundreds of thousands of molecules. This workflow (see Figure 6) processes each molecule in the same manner as the non-iterative descriptor calculation workflow but it uses different database workers. Instead of the single database worker Get_Molecules_From_Database three database workers are applied: Iterative_Molecule_From_Database_Reader, Get_Molecule_From_Database and Has_Next_Molecule_From_Database. The first of the three workers is used to configure the database connection and store it within an internal object registry. The second worker gets the ID of the database connection as an input and loads molecules from the database. Only a subset of the original query is loaded using the SQL functions LIMIT and OFFSET. The last database worker checks whether the set of loaded molecules is the last of this query or if further molecules must be loaded. If the latter applies the output of this last worker would be the text value true. A last but essential worker is Fail_if_true. This worker throws an exception if it gets the value true as input. This worker is crucial for the nested workflow: If it fails the whole nested workflow fails. Taverna then provides a retry mechanism for a failing worker or nested workflow. This mechanism is used to re-run the nested workflow as often as necessary. This dirty workaround might become obsolete in later versions of Taverna.

#### Scenario 4: Validation of CDK Atom Types

**Figure 7:**
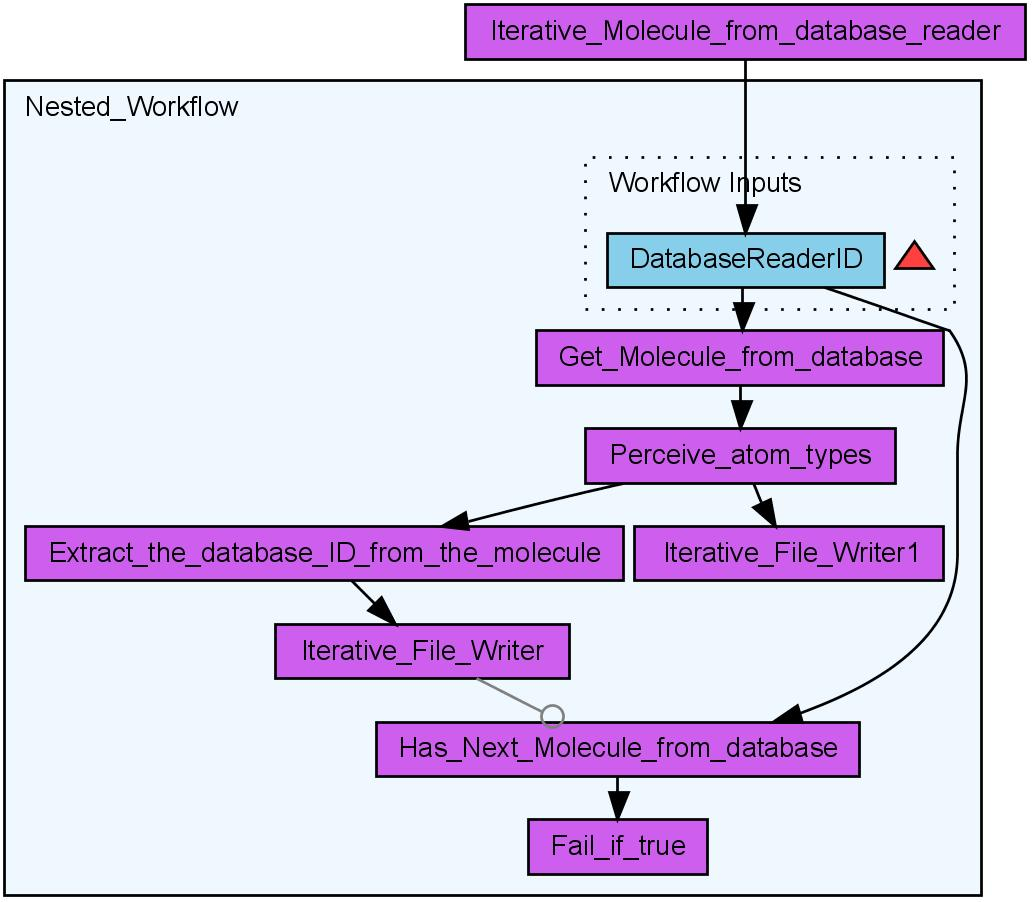
**Workflow for iterative loading of molecules from a database and searches for molecules with atom types unknown to the Chemistry Development Kit (Scenario 4)**.

**Figure 8:**
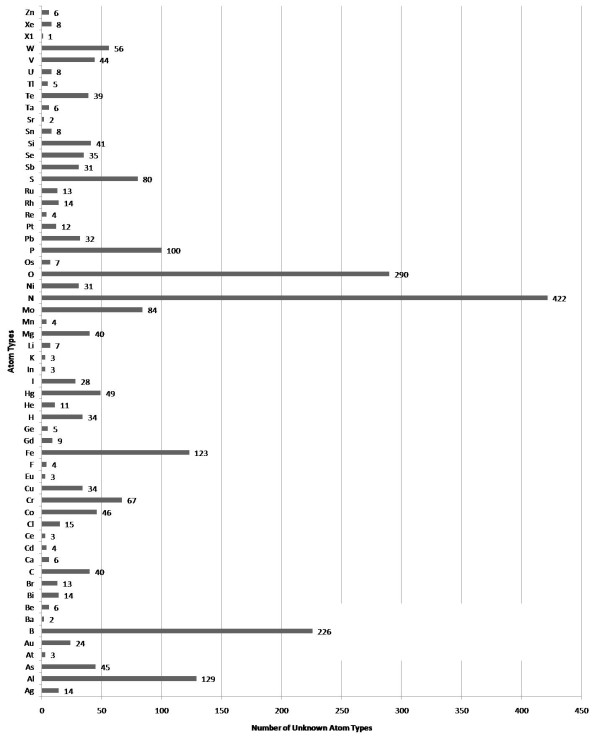
**Allocation of the unknown atom types detected during the analysis of the ChEBI database (12367 molecules)**. A total of 2414 atoms in 1035 molecules (8.36%) did not have a recognized atom type. X1 summarizes unrecognized atom types for the elements Am, Cf, Cm, Dy, Es, Fm, Ga, Lr, Md, Na, Nb, No, Np, Pm, Pu, Sm, Tb, Tc, Th, and Ti.

The calculation of physicochemical properties in the Chemistry Development Kit (CDK) relies on the detection of atom types for all atoms in each molecule. The atom types describe basic atomic properties needed by the various cheminformatics algorithms implemented in the CDK. If no atom type is recognized for an atom, the atom is flagged as *unknown*. Based on the CDK's atom type perception functionality, we devised an example workflow (see Figure 7) for the validation of the CDK atom typing procedures. The detection of an unknown atom type by the CDK indicates that either the CDK lacks this specific atom type or the molecule contains chemically nonsensical atom types. In Figure 7 the Perceive_atom_types worker performs an atom type detection, followed by the retrieval of the database ID for those molecules with unknown atom type by the Extract_the_databaseID_from_the_molecule worker. The workflow creates two text files, one containing the identifier of all molecules with unknown atom types, created by the Iterative_File_Writer, and a second one containing information about which atom of which molecule is unknown to the CDK. An analysis [23] of the atom type detection was performed on three different databases, two proprietary natural products databases and the open access database of Chemical Entities of Biological Interest (ChEBI) [32,33], maintained at the European Bioinformatics Institute (EBI). The workflow was run with more than 600 thousand molecules and showed that the CDK algorithms matches the atom types quite well, but that the atom type list is not complete for metals and other heavy atoms (see Figure 8). Missing atom type definitions is a general problem to many cheminformatics algorithms and not unique to the CDK: it leads to severe problems and computation error. Therefore, initial atom type perception is an important filter for cheminformatics workflows.

#### Scenario 5: Reaction Enumeration

Markush structures are chemical drawings which represent a series of molecules by indicating locations where differences occur. These locations are marked as *Heterocyclic*, *Alkyl*, or identified by an *R *group, enumerating a series of possible groups, such as *Methyl*, *Isopropyl*, and *Pentyl*. Markush structures are commonly used in patents for describing whole compound classes and are named after Eugene A. Markush who described these kind of structures firstly in his US patent in the 1920s.

In the process of reaction enumeration, Markush structures are used to design generic reactions. These reactions are usable for the enumeration of large chemical spaces, which includes the generation of chemical target libraries. The results of the enumeration have important applications in patent formulation and in High Throughput Screening (HTS). HTS experiments screen large amounts of small molecules, called molecule libraries, against one or more assays for testing for biological activity. A couple of years ago, the libraries used for a single HTS experiment consisted of up to 100.000 molecules. Nowadays, more targeted libraries of a reduced size of up to 1.000 molecules are used, but still commonly defined using Markush structures.

**Figure 9:**
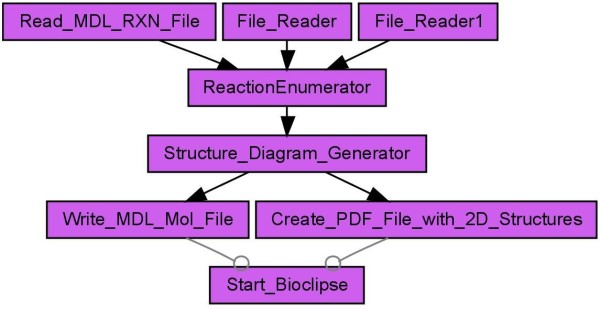
**Reaction enumeration (Scenario 5) loading a generic reaction from a MDL RXNfile and two reactant lists from MDL SDfiles**. The products from the enumeration are stored as MDL Molfiles. Besides these files a PDF document showing the 2D structure of the products is created. At the end Bioclipse will start up to allow visualization and analysis of the results [39].

**Figure 10:**
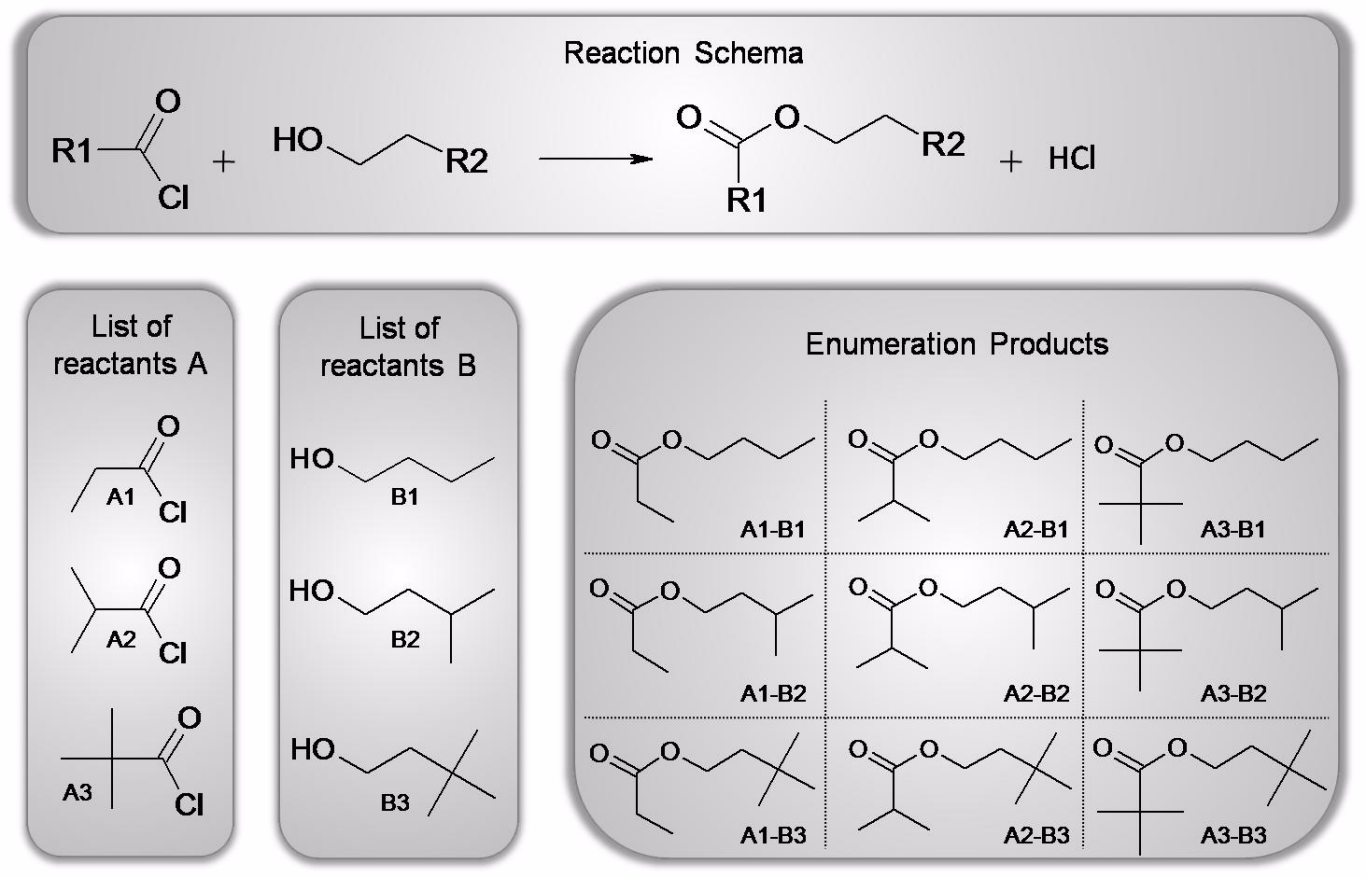
**Reaction enumeration example with two building blocks**. For each building block, a list of three reactants is defined. This enumeration results in nine different products.

For reaction enumeration, a given reaction contains different building blocks, which are needed for the enumeration. Each reactant of the reaction represents a building block. A scientist then selects a number of molecules for each reactant and the reaction enumeration creates a list of all possible products. The list of products then passes a virtual screening before at last a scientist decides which products will be synthesized. Results can be visualized and inspected at the end in Bioclipse [17]. CDK-Taverna contains workers which support an enumeration task based on a generic reaction (see Figures 9 and 10).

#### Scenario 6: Clustering Workflows

**Figure 11:**
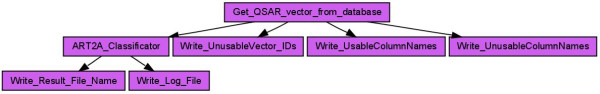
**Workflow for loading molecular descriptor data vectors from a database, followed by a ART 2-A clustering (Scenario 6)**.

During the work on this project the majority of workflows used unsupervised clustering with an implementation of the ART 2-A algorithm [34]. This algorithm was chosen because of its capability to automatically cluster open-categorical problems. Compared to traditional clustering methods like the k-means, the ART 2-A algorithm is computationally less demanding and therefore applicable especially to large data sets. Within a typical clustering workflow the Get_QSAR_vector_from_database worker loads molecule's data vectors (compare descriptor calculation workflow above) for a specific molecular SQL query from the database. This worker provides options to inspect the result vector which includes checks for values such as Not a Number (NaN) or Infinity. In addition, different thresholds may be specified for components or complete vector removal, e.g. for the removing of components whose minimum value equals its maximum value. After the loading and cleaning of a data vector, the clustering task is performed using the ART2A_Classificator worker, as depicted in Figure 11. For the configuration of this worker different options are available:

• linear scaling of the input vector to values between 0 and 1,

• a switch between *deterministic random *and *random random *for the selection of the vectors to process,

• the definition of the convergence criteria of the clustering process,

• the required similarity for the convergence criteria,

• the maximum clustering time, and

• a limit for the number of clustering steps and a range for the vigilance parameter that guides the ART 2-A algorithm.

**Figure 12:**
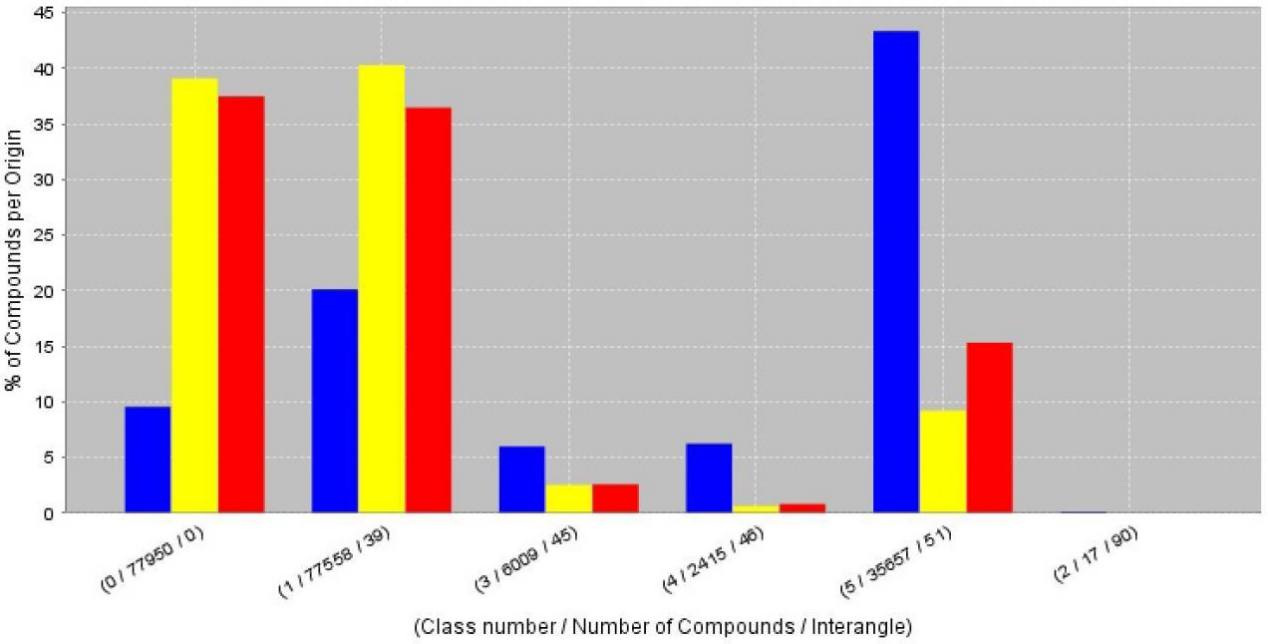
**Occupancies of six different detected clusters for two proprietary natural product databases (yellow and red) with the ChEBI database (blue), highlighting the unique character of the ChEBI database**.

The implemented ART 2-A algorithm contains two possible convergence criteria. It converges if the classification does not change after one epoch or if the scalar product of the classes between two epochs is less than the required similarity. The clustering result is stored in form of a compressed XML document. This XML result document can be processed with different workers to create different visualizations depending on the aim of the clustering task. For chemical diversity analysis the ART 2-A worker was used for a successive top-down clustering of three chemical databases (two proprietary databases containing natural products and the ChEBI database [33] containing molecules of biological interest, see Figure 12). The occupancies of the different clusters show the similarity of the natural product databases in contrast to the ChEBI database which differs in chemical space occupation [23]. This findings will be outlined in a subsequent publication.

## Conclusions

With CDK-Taverna we have presented the first free and open cheminformatics workflow solution for the biosciences. It allows to link and process data from various sources in visually accessible workflow diagrams without any deeper programming experience. Processing of hundreds of thousands of molecules has been demonstrated and the upper boundary is only limited by the amount of available memory. The currently implemented workers allow the processing of chemical data in various formats, provides the possibility to calculate chemical properties and allows cluster analysis of molecular descriptor vectors. The use of the PostgreSQL database with the Pgchem::tigres cheminformatics cartridge provides access to chemical databases with up to a million molecules.

## Availability and requirements

• **Project name**: CDK-Taverna

• **Project home page**: http://www.cdk-taverna.de

• **Operating system(s)**: Platform independent

• **Programming language**: Java

• **Other requirements**: Java 1.6.0 or higher http://java.sun.com/, Taverna 1.7.2 http://sourceforge.net/projects/taverna/files/taverna/1.7.2/

• **License**: GNU Library or Lesser General Public License (LGPL)

• **Any restrictions to use by non-academics**: none

## Authors' contributions

EW initiated the integration of Taverna and the CDK. CS and AZ conceived the project, and lead the further development. TK did the majority of CDK-Taverna development and developed the projects to its current state. All co-authors contributed to the manuscript. All authors read and approved the final manuscript.
